# Morphological risk factors associated with dislocation after bipolar hemiarthroplasty of the hip in patients with femoral neck fractures—a nested case-control study

**DOI:** 10.1186/s13018-019-1409-1

**Published:** 2019-11-28

**Authors:** Yueqi Zhang, Zhenjun Yao, Peng Shi, Chenzhong Wang, Jinyu Liu, Yi Yang, Chi Zhang

**Affiliations:** 1 Department of Orthopedic Surgery, Zhongshan Hospital, Fudan University, 180 Fenglin Road, Shanghai, 200032 China; 2 Children’s Hospital of Fudan University, 399 Wanyuan Road, Shanghai, 201102 China

**Keywords:** Hemiarthroplasty, Dislocation, Acetabular depth, Center-edge angle

## Abstract

**Background:**

The relationship between preoperative hip measurements and dislocation after bipolar hemiarthroplasty is presently unclear. In the current study, we investigated the morphological risk factors associated with dislocation after bipolar hemiarthroplasty of the hip in patients with femoral neck fractures.

**Methods:**

Between January 2011 and June 2017, a nested case-control design study was used to analyze the risk factors for dislocation in 348 patients who had undergone bipolar hemiarthroplasty because of femoral neck fractures. Twelve patients underwent at least one dislocation postoperatively. Sixty patients without dislocation were selected as controls matched in terms of time of surgery, age, and sex, at a ratio of 1:5. Patient acetabular measurements were compared between the dislocation group and the control group, including the center-edge angle, abduction angle, acetabular width and depth, depth-to-width ratio, femoral neck offset, leg length discrepancy, and femoral head coverage ratio. A multivariate logistic regression model was used to evaluate the morphological risk factors of dislocation.

**Results:**

Postoperatively, the incidence of dislocation was 3.4%. A smaller center-edge angle was found to be a risk factor associated with dislocation after bipolar hemiarthroplasty of the hip. Patients with small acetabular depth and a small acetabular depth–width ratio were prone to dislocation. Patients with a center-edge angle of ≤ 45.4° or an acetabular depth of ≤ 19.12 mm were more likely to suffer dislocation.

**Conclusions:**

Careful preoperative measurements before bipolar hemiarthroplasty of the hip are important. Surgical intervention for femoral neck fracture patients with a shallow acetabulum should be carefully planned and total hip arthroplasty should be considered when necessary.

## Background

Femoral neck fracture is one of the most common orthopedic fractures in the elderly, which directly influences their mobility and causes clinical complications leading to a higher mortality rate [1, 2]. With the aging population, it is predicted that the total number of femoral neck fractures will rise to 6.26 million per year in 2050 [3]. Surgical treatment options for femoral neck fractures include internal fixation, hemiarthroplasty or total hip arthroplasty (THA) [4]. A study by Haidukewych et al. showed that bipolar hemiarthroplasty was associated with excellent component survivorship in elderly patients with displaced femoral neck fractures [5]. A bipolar hemiarthroplasty has become the accepted treatment for elderly displaced femoral neck fracture patients. Compared with THA, bipolar hemiarthroplasty has the advantages of a lower economic burden and a lower risk of dislocation after surgery [6]. Dislocation after bipolar hemiarthroplasty of the hip is a rare but devastating complication that has a great impact on patient quality of life [7, 8].

The specific risk factors for dislocation have not yet been identified but generally can be classified into patient factors, surgical factors, and morphological factors. Patient factors (history of neurological disease and weakness of abduction muscles), morphological factors (center-edge angle, femoral neck offset, and leg length discrepancy), and surgical factors (surgery approach, choice of prosthesis, and repair of the short external rotator tendons) were reported to play an important role in dislocation after bipolar hemiarthroplasty [9–12]. Adanir et al. reported measurements such as the center-edge angle and acetabular depth (which evaluated the shallowness of the acetabulum) were used to assess acetabular dysplasia [13], but the relationship between these measurements and dislocation after bipolar hemiarthroplasty for patients without acetabular dysplasia remained unclear. In the current study, we explored the morphological risk factors for dislocation after bipolar hemiarthroplasty.

## Materials and methods

Between January 2011 and June 2017, 348 patients underwent bipolar hemiarthroplasty in our department because of femoral neck fractures. A total of 350 bipolar hemiarthroplasties were performed with 2 patients who underwent bilateral surgeries. All patients had a minimum follow-up of at least 1 year.

**Fig. 1:**
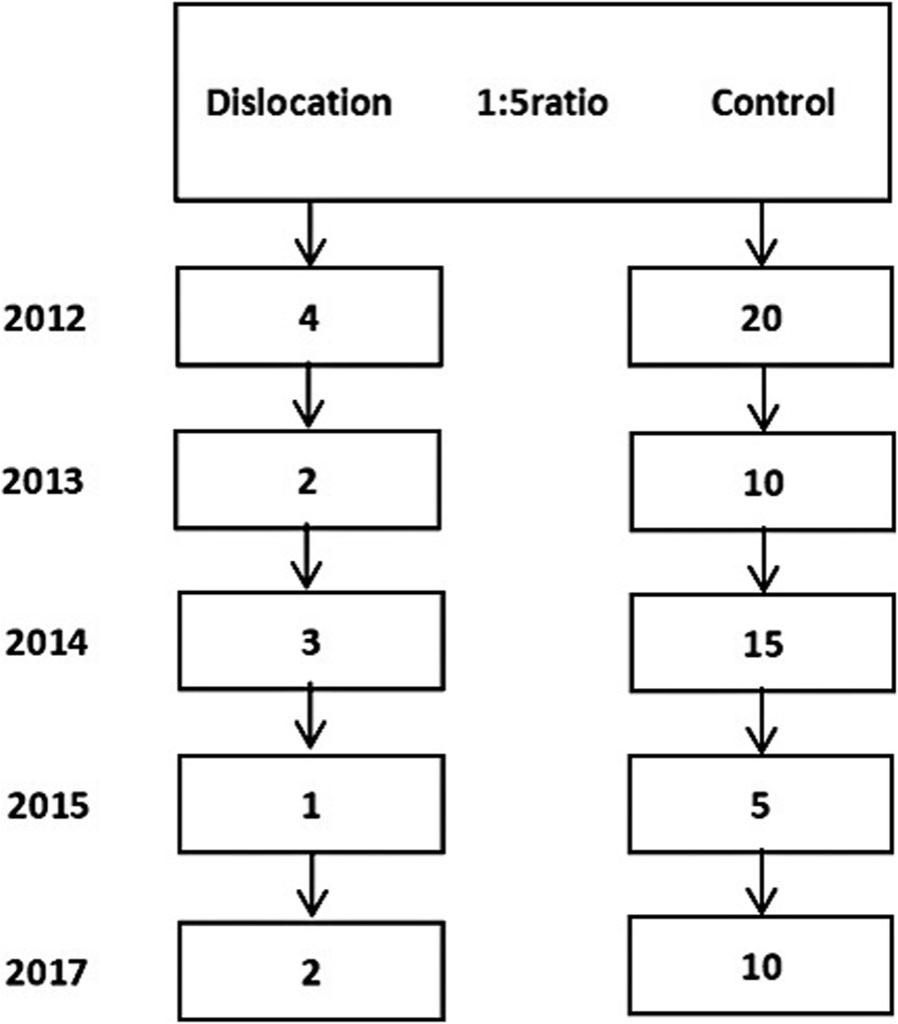
The flowchart showed a nested case-control study with a ratio of 1:5 during the follow-up time from 2011 to 2017

A nested case-control design was used to analyze the risk factors for dislocation. Dislocation occurred in 12 patients and 2 patients experienced more than 1 incident of dislocation. A control group of 60 patients without dislocation were selected from the remaining 336 patients, based on age, sex, and time of surgery (approximate ratio of 1:5 of case to control), as shown in Fig. 1. Seven patients with dislocation were treated through closed reduction while three patients were treated through open reduction. Two patients who had recurrent dislocations were treated through conversion to THA.

All bipolar hemiarthroplasties of the hip were performed following a posterolateral approach. Postoperative standard hip anteroposterior and lateral radiographs were obtained and rehabilitation training was guided by the same rehabilitation therapist team for all patients after surgery. All patients were instructed to avoid excessive hip adduction and internal rotation. Weight-bearing exercises started with the help of a walker or crutches during the first week and gradually increased to full weight-bearing within 4 weeks after surgery.

Patient data (age, sex, medical history including dementia, Parkinson’s disease, lacunar infarction, and diabetes) were collected from patient medical records. Morphological factors, including center-edge angle, abduction angle, acetabular depth and width, depth-to-width ratio, femoral neck offset, leg length discrepancy, and femoral head coverage ratio were measured from the hip anteroposterior radiographs after surgery.

**Fig. 2:**
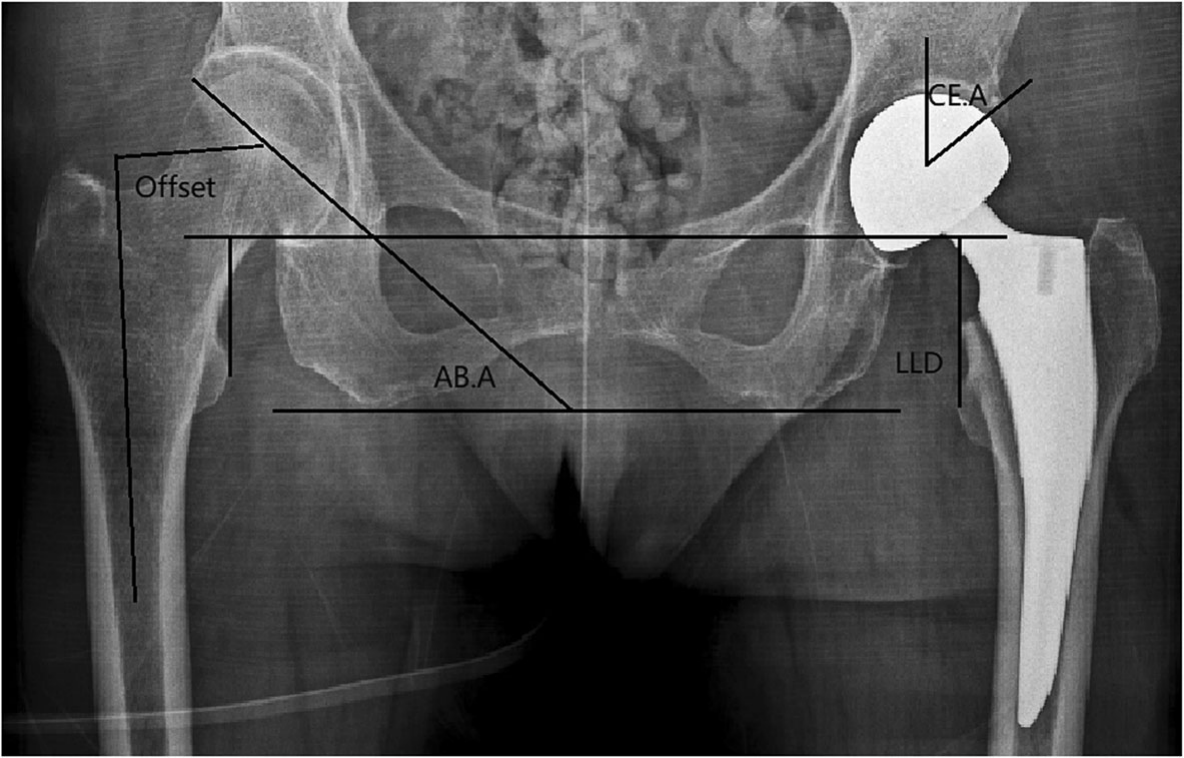
Measurement of center-edge angle (CE.A), abduction angle (AB.A), leg length discrepancy (LLD), and femoral neck offset. (LLD is the difference in perpendicular distance between a line passing through the lower edge of the teardrop points to the corresponding tip of the lesser trochanter)

**Fig. 3:**
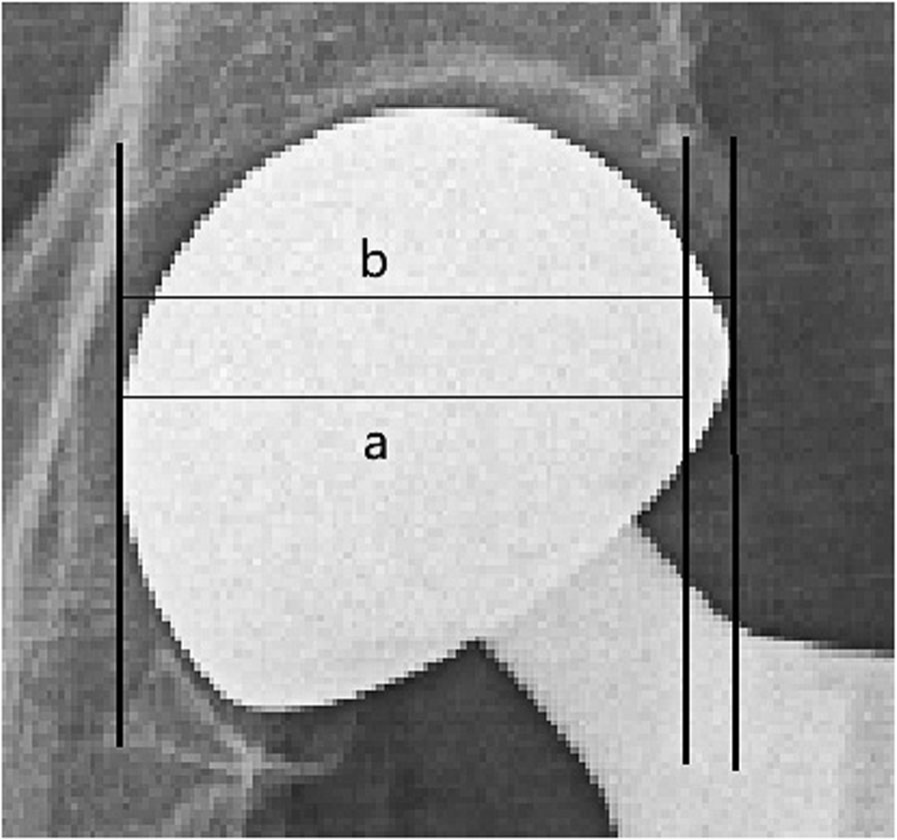
Femoral head coverage ratio: ratio of the length between the innermost point of the femoral head and the outer corner of the acetabulum to the length of the femoral head (ratio of a to b)

**Fig. 4:**
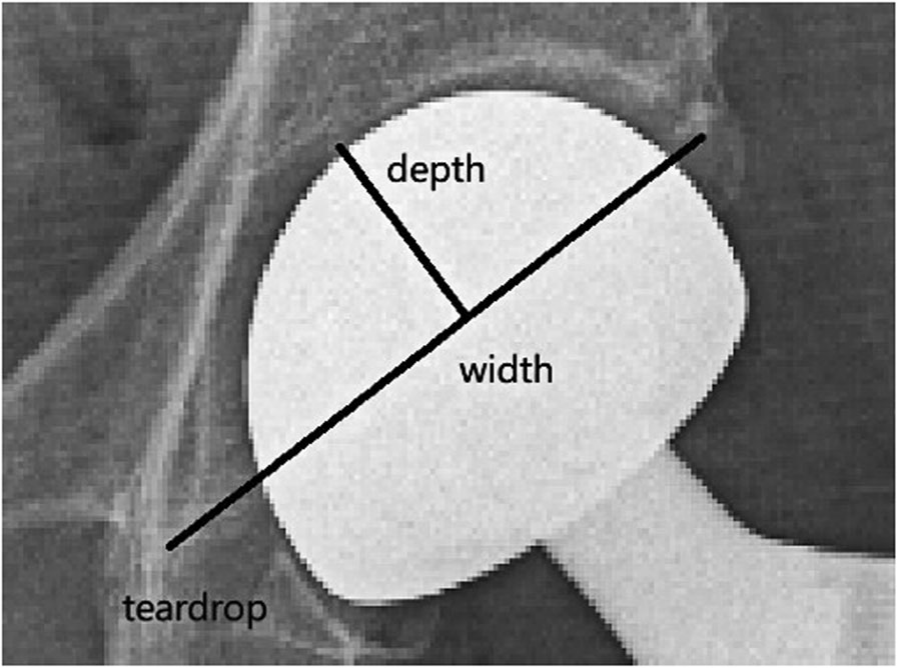
Acetabular width: length of the line joining the lateral edge of the acetabulum to the pelvic teardrop. Acetabular depth: length of another line perpendicular to width line at the point of the greatest acetabular depth. Depth to width ratio: ratio of acetabular depth to width

The measurements of the center-edge angle (CE angle), abduction angle femoral neck offset, and leg length discrepancy are shown in Fig. 2 [14–16]. The measurement of the femoral head coverage ratio is shown in Fig. 3 [13]. The measurement of the acetabular width, depth, and depth to width ratio are shown in Fig. 4 [17].

## Statistical analysis

### Statistical methods

Categorical data were presented as counts and percentages. Continuous variables were presented as mean ± SD or median (P25, P75) according to their distributions. The chi-square test was applied to test the equality of proportions. The Student *t* test or Mann-Whitney *U* test was used to test the difference between the two groups. A logistic regression analysis was performed to evaluate the hip measurements associated with dislocation. A receiver operating characteristic (ROC) curve was used to predict certain factors related to dislocation. In all the tests, a *P* value of < 0.05 was considered to indicate statistical significance. All the statistical analyses were performed using SPSS19.0 (SPSS Inc., Chicago, IL, USA). ROC curves were created using MEDCALC software (MedCalc Software, Mariakerke, Belgium).

## Results

The incidence of dislocation in the current study was 3.4% (12 of 348 patients), 1 male and 11 female patients with a mean age of 85 years. The value of the time from fracture to surgery and the time from surgery to dislocation followed the non-normal distribution, while all the other variables including the hip measurements followed the normal distribution according to the normal distribution test. All the hip measurements followed the equality of the variance between the two groups according to Levene’s test (*P* > 0.10). The median time from bipolar hemiarthroplasty to the first incident of dislocation was 36 days (range, 3–137 days). Eleven dislocations occurred within 2 months after surgery and 1 at 137 days. Eight patients suffered dislocations without trauma while four patients suffered dislocations because of a same-level fall (Table 1).

**Table 1:** Dislocation patient demographics including the sex, age, time from surgery to dislocation, cause of dislocation, treatment method, and medical history

Patient number	Sex	Age (years)	Time from surgery to first dislocation (days)	Cause of dislocation	Treatment	Parkinson or dementia
1	Female	80	46	Fall	Closed reduction	No
2	Female	87	15	Atraumatic	Closed reduction	Dementia
3	Female	85	31	Fall	Closed reduction	No
4	Female	75	15	Atraumatic	Open reduction	No
5	Female	88	15	Fall	Closed reduction	No
6	Male	83	23	Fall	Open reduction	Parkinson
7	Female	89	54	Atraumatic	Closed reduction	No
8	Female	84	14	Atraumatic	Open reduction	No
9	Female	88	137	Atraumatic	Closed reduction	No
10	Female	84	43	Atraumatic	Closed reduction	No
11	Female	84	3	Atraumatic	THA	Parkinson
12	Female	90	30	Atraumatic	THA	No

No statistically significant difference existed between the two groups regarding age, sex, and medical history, including Parkinson’s disease, dementia, lacunar infarction, and diabetes. The median time from fracture to surgery was 4 (range, 0.5–7) days in the dislocation group and 3 (range, 2–6) days in the control group, with no significant difference according to the Mann-Whitney *U* test (Table 2).

**Table 2:** Comparison of clinical data and acetabular measurements between dislocation and control group

Characteristics	Dislocation *n* = 12	Control *n* = 60	*P* value
Age, years, mean ± SD	84.8 ± 4.2	84.6 ± 4.5	0.897
Sex, *n* (%)			
Male	1(8.3)	5(8.3)	
Female	11(91.7)	55(91.7)	
Disease, *n* (%)			
Parkinson’s	2(16.7)	4(6.7)	0.567
Dementia	1(8.3)	2(3.3)	0.426
Lacunar infarction	5(41.7)	23(38.3)	1.000
Diabetes	1(8.3)	14(23.3)	0.436
Time from fracture to surgery days , median (P25, P75)	4(0.5,7)	3(2.6)	0.744
Measurements of hip, mean ± SD			
CE.A,°	38.9 ± 5.9	48.7 ± 5.4	< 0.001
AB.A,°	37.9 ± 3.1	34.2 ± 3.7	0.002
Width, mm	64.16 ± 3.03	63.25 ± 4.07	0.520
Depth, mm	17.58 ± 2.19	20.41 ± 1.73	< 0.001
Offset, mm	31.88 ± 8.09	33.23 ± 7.35	0.569
LLD, mm	2.77 ± 8.43	4.94 ± 6.53	0.322
D/W	0.28 ± 0.036	0.32 ± 0.027	< 0.001
FC.R	0.87 ± 0.043	0.92 ± 0.047	< 0.001

*CE.A* center-edge angle, *AB.A* abduction angle, *LLD* leg length discrepancy, *D/W* ratio of acetabular depth to width, *FC.R* femoral head coverage ratio

Compared with an age-and-sex-matched control group, five hip measurements showed statistically significant differences in the dislocation group according to the Student *t* test, as shown in Table 2. Patients in the dislocation group had a smaller CE angle (38.9 ± 5.9° vs. 48.7 ± 5.4°, *P* < 0.001), a greater abduction angle (37.9 ± 3.1 vs. 34.2 ± 3.7°, *P* = 0.002), a smaller acetabular depth (17.58 ± 2.19 mm vs. 20.41 ± 1.73 mm, *P* < 0.001), a smaller depth-to-width ratio (0.28 ± 0.036 vs. 0.32 ± 0.027, *P* < 0.001), and a smaller femoral head coverage ratio (0.87 ± 0.043 vs. 0.92 ± 0.047, *P* < 0.001) (Table 2). There were no statistically significant differences in other hip measurements according to the Student *t* test, as shown in Table 2.

To investigate the five measurements (CE angle, abduction angle, acetabular depth, depth-to-width ratio, and femoral head coverage ratio), which demonstrated significant differences in the dislocation group, we used a multivariate logistic regression model. We formulated three models to investigate the link between the acetabular depth and depth-to-width ratio (Table 3). To compare their effects on the risk of dislocation, the values of the depth-to-width ratio, acetabular depth, and femoral head coverage ratio were standardized into D/W-z, D-z, and FC.R-z using *z*-score normalization. A significant difference in the CE angle was observed in all three models. In model 1, which involved all five measurements, the CE angle demonstrated the most significant difference (OR, 0.726; 95%CI, 0.529–0.996; *P* = 0.047). In model 2, where depth (D-z) was analyzed instead of depth-to-width ratio (D/W-z), a decreased CE angle (0R, 0.739; 95%CI, 0.556, 0.982; *P* = 0.037) and a decreased depth (D-z) (OR, 0.146; 95%CI, 0.025–0.860; *P* = 0.033) were found to increase the risk of dislocation. In model 3, where depth-to-width ratio (D/W-z) was analyzed instead of depth (D-z), D/W-z showed the same trend as D-z (OR, 0.076; 95CI%, 0.004–1.048; *P* = 0.084), and additional effects were associated with a *P* value approaching significance. The depth-to-width ratio (D/W-z) may have more predictive value than depth (D-z).

**Table 3:** Result of logistic regression of 5 measurements in 3 models

Variables	Model 1		Model 2		Model 3	
	OR(95%CI)	*P*	OR(95%CI)	*P*	OR(95%CI)	*P*
CE.A	0.726(0.529, 0.996)	0.047	0.739(0.556, 0.982)	0.037	0.708(0.509, 0.986)	0.041
AB.A	0.876(0.626, 1.227)	0.442	0.918(0.688, 1.226)	0.564	0.851(0.614, 1.179)	0.331
FC.R-z	0.874(0.234, 3.267)	0.841	0.764(0.233, 2.502)	0.657	0.859(0.244, 3.028)	0.813
D-z	0.493(0.025, 9.745)	0.642	0.146(0.025, 0.860)	0.033		
D/W-z	0.170(0.002, 12.106)	0.416			0.076(0.004, 1.408)	0.084

*CE.A* center-edge angle, *AB.A* abduction angle, *FC.R-z* femoral head coverage ratio-z, *D-z* acetabular depth-z, *D/W-z* depth-to-width ratio-z

**Fig. 5:**
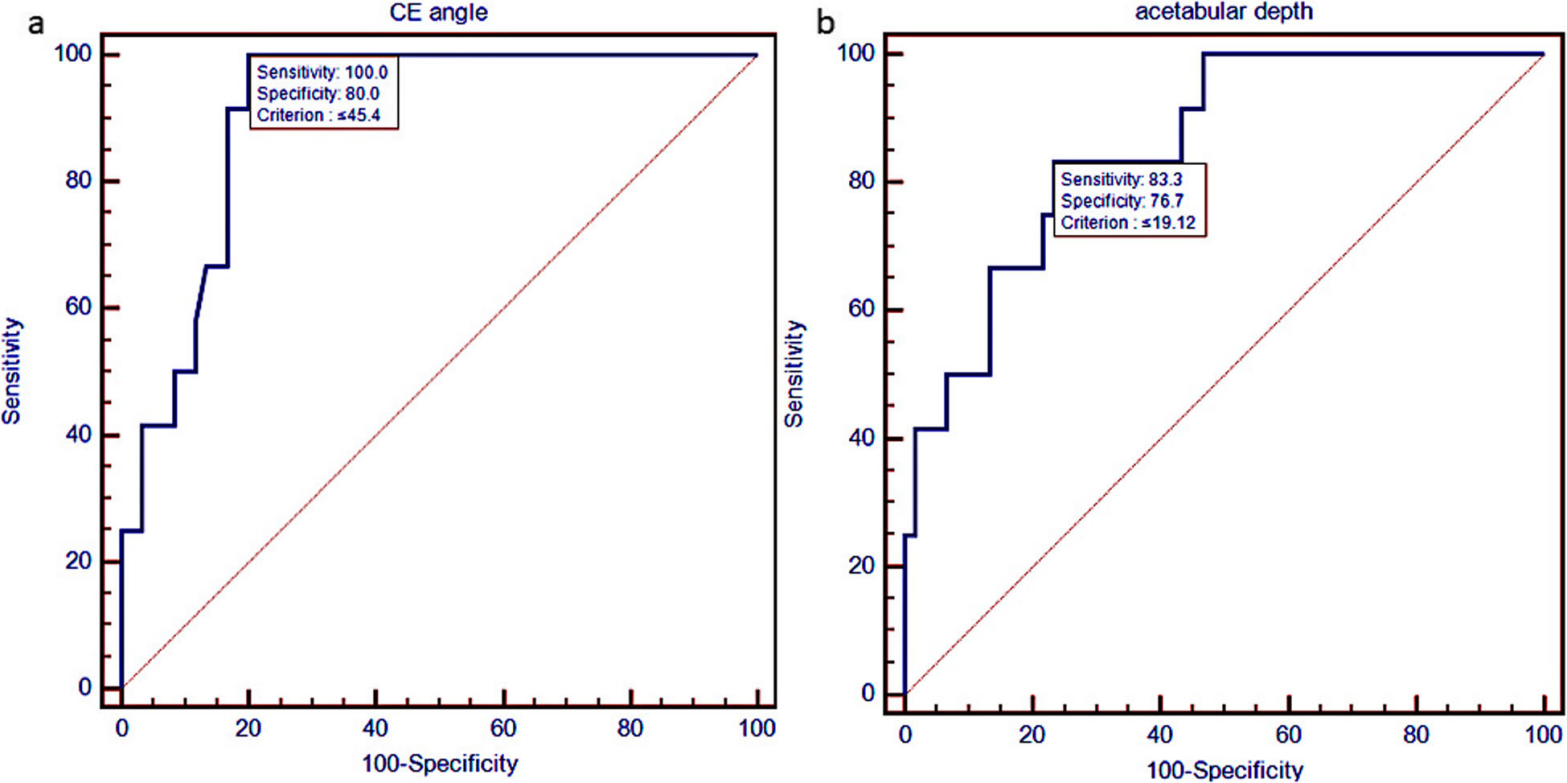
ROC curves of **a** CE angle and **b** acetabular depth as criterion for dislocation

The abduction angle and femoral head coverage ratio both showed no significant difference in all three models. A smaller acetabular depth was associated with a higher risk of dislocation (OR, 0.146; 95%CI, 0.025–0.860; *P* = 0.033) (model 2). However, when the depth-to-width ratio was considered (model 1), it was not statistically significant (OR, 0.493; 95%CI, 0.025–9.745; *P* = 0.642). As shown in the ROC curve, predicting dislocation using the existence of a CE angle of no more than 45.4° had a sensitivity of 100% and specificity of 80% (Fig. 5a). In addition, predicting dislocation using the existence of an acetabular depth of no more than 19.12 mm had a sensitivity of 83.3% and a specificity of 76.7% (Fig. 5b).

## Discussions

It has been reported that compared with THA, bipolar hemiarthroplasty has a lower dislocation rate after surgery [6]. In theory, bipolar hemiarthroplasty involves an additional articulating joint within the head, thereby allowing movement to occur both at the prosthesis acetabular interface and within the prosthesis. In addition, the metallic shell has a large diameter, reducing the dislocation rate [18]. However, in the current study, dislocation still occurred at a rate of 3.4%, which is in agreement with the published incidence of dislocation ranging from 1.1 to 5% [5, 19, 20]. The factors contributing to dislocation are still controversial. Many factors have been reported to be associated with the occurrence of dislocation such as neuromuscular disease, surgical approach and the choice of prosthesis [9–12]. We performed the current study to investigate if morphological factors are associated with dislocation after bipolar hemiarthroplasty. We found that most of the dislocation patients had smaller acetabular coverage compared with the control group patients.

Radiographically, acetabular dysplasia is defined by either a CE angle of ≤ 25° (severe if ≤ 20°) or an acetabular depth of < 9 mm [21, 22]. In the current study, all the dislocation patients had a CE angle ranging from 29.6° to 45.4°, and an acetabular depth ranging from 13.03 mm to 20.65 mm, indicating that they did not display acetabular dysplasia. Although the patients were not considered to have acetabular dysplasia, the ROC curve showed that patients with a CE angle of ≤ 45.4° or an acetabular depth of ≤ 19.12 mm were more likely to suffer dislocation after bipolar hemiarthroplasty. A Korean study also reported that people with a CE angle of ≤ 44° were significantly more likely to suffer dislocation compared with those with a CE angle of >44° [10]. Here, we studied the ROC curve for the CE angle as well as for the acetabular depth.

In the current study, we found that a smaller CE angle was a major risk factor for dislocation after bipolar hemiarthroplasty (OR, 0.726; 95%CI, 0.529–0.996; *P* = 0.047, model 1, Table 3). People with a smaller acetabular depth were more likely to suffer dislocation (OR, 0.46; 95%CI, 0.025–0.860; *P* = 0.033, model 2, Table 3). Acetabular depth can directly indicate the shallowness of the acetabulum and may affect dislocation. Compared with absolute acetabular depth, a smaller depth-to-width ratio may better describe the shallowness of the acetabulum in the case that a small acetabular width is also present. Although the depth-to-width ratio showed no significant difference in all the models, it should be taken into consideration when the acetabulum is measured. Compared with acetabular depth, the depth-to-width ratio may reduce individual variation and better evaluate the degree of shallowness of the acetabulum (Table 3).

Bipolar hemiarthroplasty does not change the morphology of the acetabular side, especially when patients have shallower acetabular measurements such as a smaller CE angle and a smaller acetabular depth, which predisposes them to dislocation. Patients with a shallower acetabulum may be prone to dislocation after bipolar hemiarthroplasty. Compared with bipolar hemiarthroplasty, THA has the advantage of adjusting the acetabular side. Cobb et al. found that when an elevated liner of the acetabulum was used in THA, the stability of the hip after THA was improved and a lower dislocation risk was found [23]_._ Nakashima Y et al. reported that cement-free stem anteversion varied widely and a combined anteversion technique is useful to reduce the incidence of dislocation in cement-free THA [24]. THA has the advantages of correcting the shallowness of the acetabulum directly by setting an acetabular cup and adjusting the combined anteversion by controlling both femoral and acetabular anteversion while bipolar hemiarthroplasty can only adjust the anteversion of the femoral side.

For recurrent dislocation after bipolar hemiarthroplasty, conversion to THA is usually used. Sierra et al. suggested that for dislocation patients with additional contributing factors such as a shallow acetabulum socket, open reduction is needed, with or without component revision including conversion to THA [20]. In a study performed by Encoson et al., 1 of 720 patients had recurrent dislocation after bipolar hemiarthroplasty because of a shallow acetabular socket and was finally treated by converting to THA [9]. In the current study, two patients suffered from recurrent dislocation. They had indications of a comparatively shallow acetabulum (CE angle of 30.3° and 31°, acetabular depth of 15.31 mm and 13.03 mm). Both of the patients were treated with a surgical conversion to THA and did not suffer from subsequent dislocations. THA has the advantage of providing better acetabulum coverage to correct acetabular shallowness.

There are some limitations to the current study. The sample size was relatively small and the incidence of dislocation was 3.4%. A nested case-control is often used when the outcome is rare or when the interest of exposure difficult to obtain. In the current study, we performed a 1:5 ratio nested case-to control study. For each dislocation patient, we matched five control patients in terms of age, sex, and time of surgery to eliminate several confounding factors and increase the reliability of the study.

## Conclusion

In conclusion, the current study suggests that a smaller CE angle is a risk factor for dislocation after bipolar hemiarthroplasty. There was a trend for people with a smaller acetabular depth or depth-to-width ratio to suffer from postoperative dislocation. A bipolar hemiarthroplasty should be carefully considered when the acetabulum tends to be shallow and a total hip replacement should be performed if necessary.

## Data Availability

The datasets used and/or analyzed during the current study are available from the corresponding author on reasonable request.

## Abbreviations

CE: Center-edge angle
D/W: Depth-to-width ratio
FC.R: Femoral head coverage ratio
ROC: Receiver operating characteristic
THA: Total hip arthroplasty
